# Geo-referenced simulation of pharmaceuticals in whole watersheds: application of GREAT-ER 4.1 in Germany

**DOI:** 10.1007/s11356-020-12189-7

**Published:** 2021-01-07

**Authors:** Volker Lämmchen, Gunnar Niebaum, Jürgen Berlekamp, Jörg Klasmeier

**Affiliations:** Institute of Environmental Systems Research, Barbarastr. 12, 49076 Osnabrück, Germany

**Keywords:** Geo-referenced modeling, Environmental fate, Pharmaceuticals, Exposure assessment, River basin management, GREAT-ER model

## Abstract

The geo-referenced regional exposure assessment tool for European rivers (GREAT-ER) is designed to support river basin management or the implementation process within the EU Water Framework Directive by predicting spatially resolved exposure concentrations in whole watersheds. The usefulness of the complimentary application of targeted monitoring and GREAT-ER simulations is demonstrated with case studies for three pharmaceuticals in selected German watersheds. Comparison with monitoring data corroborates the capability of the probabilistic model approach to predict the expected range of spatial surface water concentrations. Explicit consideration of local pharmaceutical emissions from hospitals or private doctor’s offices (e.g., for X-ray contrast agents) can improve predictions on the local scale without compromising regional exposure assessment. Pharmaceuticals exhibiting low concentrations hardly detectable with established analytical methods (e.g., EE2) can be evaluated with model simulations. Management scenarios allow for a priori assessment of risk reduction measures. In combination with targeted monitoring approaches, the GREAT-ER model can serve as valuable support tool for exposure and risk assessment of pharmaceuticals in whole watersheds.

**Supplementary Information:**

The online version contains supplementary material available at 10.1007/s11356-020-12189-7.

## Introduction

A major problem for humankind is access to clean and readily available drinking water. Therefore, protection of groundwater and surface water against unwanted and potentially harmful chemical contaminants is important. The European Water Framework Directive (WFD) constitutes a legal framework that imposes the protection of common water resources on European states (EU 2000). The call of the directive among other things is the good chemical status of European surface waters. To achieve this goal, exposure and risk assessment of micropollutants, including pharmaceuticals, followed by development and implementation of reduction measures for critical compounds is necessary. Currently, the WFD lists 45 priority substances in Annex X of the directive and sets environmental quality standards for each of these substances.

A prerequisite for the definition and implementation of mitigation measures is knowledge of the exposure concentrations of chemicals in the aqueous environment. This has led to large monitoring efforts for so-called emerging contaminants such as pharmaceuticals. To focus these efforts on potentially harmful substances, a watch list was established in 2015 whose purpose is to enforce collection of concentration data for those emerging pollutants for which available monitoring data are considered insufficient. The first watch list included diclofenac, three hormones (estrone (E1), 17β-estradiol (E2), and ethinylestradiol (EE2)), and three macrolide antibiotics (erythromycin, clarithromycin, azithromycin). The list is regularly reviewed in order to respond to new information and to avoid monitoring of substances for longer than necessary. In a recent review conducted by the Joint Research Centre (JRC) of the EU, it was concluded that diclofenac could be removed and the updated list should instead include the two antibiotics amoxicillin and ciprofloxacin among thirteen other substances (Loos et al. 2018).

In the last years, numerous papers have been published demonstrating the ubiquitous presence of pharmaceutically active substances in surface waters all over the world (e.g., Ivešić et al. 2017; Chiffre et al. 2016; Nebot et al. 2015). The monitoring data show a large variability of micropollutants’ surface water concentrations in time and space. Consequently, each data point should always be interpreted in relation to environmental conditions during sampling, e.g., values of key parameters such as river flow. However, it is obvious that permanent basin-wide monitoring of thousands of possible contaminants is virtually impossible. Moreover, even if selection of sampling sites has been done considering local circumstances, spatial variability of the monitoring results can often not be satisfyingly explained. At this point, geo-referenced simulation models can be of great help for exposure and risk assessment such as the GREAT-ER model (Kehrein et al. 2015). Other prominent examples are substance flow models set up for Switzerland (Ort et al. 2009; Kuroda et al. 2016) and the Netherlands (Coppens et al. 2015) or the LF2000-WQX water quality model (Price et al. 2010).

The well-established model GREAT-ER (Geo-referenced Regional Exposure Assessment Tool for European Rivers) predicts spatially resolved exposure concentrations for down-the-drain chemicals (Kehrein et al. 2015; Aldekoa et al. 2013; Alder et al. 2010; Koormann et al. 2006; Feijtel et al. 1998). Simulation results can be used to easily identify river sites where elevated concentrations, e.g., above a defined target value (PNEC or EQS), are expected. This information can support targeted selection of sampling sites and compliment the interpretation of monitoring data in terms of plausibility. Additionally, simulations of management scenarios for selected reduction measures and *a priori* evaluation of their effectiveness can be very helpful for water managers.

The objective of this paper is to illustrate the capabilities and limitations of GREAT-ER 4.1 using meaningful case studies for selected pharmaceuticals in three different German catchments. In particular, we demonstrate (1) the usefulness of the probabilistic model approach to consider natural variability of river flow that is reflected by the temporal variability of measured concentrations at selected sites, (2) the explicit consideration of hospital wastewater emissions important for pharmaceuticals predominantly emitted at the location of treatment, (3) basin-wide exposure assessment for substances with low PEC and EQS values, and (4) the informative value of management scenario simulations.

## The GREAT-ER 4.1 model software

### How the model works

The GREAT-ER model calculates spatially explicit steady-state concentrations of down-the-drain chemicals in surface waters of entire catchment areas considering point and non-point emissions from different sources assuming more or less constant emissions over time (Kehrein et al. 2015; Hüffmeyer et al. 2009). In general, wastewater from households, hospitals, and industry as well as runoff from agricultural areas can be taken into account as emission sources. Household emissions are treated according to the place of residence using an average per capita consumption value. In GREAT-ER 4.1, a hospital sub-model to investigate the local effect of hospital wastewater on the concentrations of selected medicinal agents has been adopted. The number of total patients (or beds) in hospitals has been suggested as appropriate proxy for respective emissions from a single hospital (Kuroda et al. 2016). Therefore, GREAT-ER 4.1 requires a per patient consumption value in this case.

The model uses mass balance equations that track the chemicals along the emission pathways into surface water including removal in wastewater treatment plants (WWTPs). Sedimentation, volatilization, and degradation by photolysis, hydrolysis, or biological processes are considered as pseudo-first-order in-stream loss processes. Mass conservation applies to each segment, so that the mass flow at the beginning corresponds to the mass flow at the end, unless it has been changed by diffuse emissions or loss processes. In the model, the river network is represented as a hydrological geometric network which is subdivided into segments (edges) of maximum length of 2000 m. Nodes are set at all confluences, point emission sites, and other points of interest (e.g., gauges, monitoring sites, weirs). Emission loads from point sources (mainly WWTPs) are estimated by a series of submodules. The loads are discharged into the receiving river at the respective node and are then transported further downstream in the model. Loads are expressed in terms of mass per unit time and are considered constant over time in order to obey to the steady-state assumption.

The model requires a number of substance-specific input parameters as well as environmental attributes. This encompasses physicochemical data, consumption, and use patterns as well as removal efficiencies during sewage treatment. The latter is modeled as simple percentage removal whose efficiency depends on the specific treatment category (lagoon, constructed wetland, bio filter, or activated sludge). Each river segment possesses a vector of attributes, e.g., flow velocity and river flow, which is used for the calculation of required intermediate parameters such as travel time. Depending on the available information, the user can choose between different complexity modes for the different submodules. A detailed description of the model equations is given in the appendix of Kehrein et al. (2015).

Natural variability of environmental parameters, uncertainty of substance parameters, and temporal fluctuation of consumption patterns can be considered by a probabilistic Monte Carlo approach. As opposed to deterministic model runs, corresponding parameters are not fixed, but defined as probability distributions of random variables. The distributions represent the expected frequency with which a parameter will take a single value. Probabilistic model runs are performed iteratively with parameter value vectors chosen from the probability distributions. The model calculates concentration distributions for each river segment mapping the expected range of the temporal variability for the selected parameter combinations. The output can be used to calculate any percentile of the respective concentration distribution. Results are primarily presented as color-coded maps or concentration profiles along a selected river course (see Figs. 2 and 4 in the case study section). In addition, a number of options for in-depth analyses of the results are implemented. Another key feature of the GREAT-ER model is the scenario builder. It enables the user to evaluate the effect of defined changes in boundary conditions on the simulated concentrations. Potential scenarios include changes in consumption, technical retrofitting of sewage treatment plants (tertiary/quaternary treatment), or re-routing of wastewater.

### How to prepare a GREAT-ER database

The GREAT-ER model core is delivered as Add-In for the commercial software ArcGIS Desktop®. The GREAT-ER philosophy follows the idea of river basin management as laid out in the EU Water Framework Directive. This means that model simulations are performed within whole catchments including all watercourses with perennial flow. All required data for the simulations must be stored in a catchment-specific database. The databases need to have a standardized structure, which is assigned during the so-called pre-processing. Here, raw data are processed to form the topological river network, to connect point sources (WWTP, industry, and hospitals), and to assign other data (gauges, monitoring sites) to the respective river segments.

Over the years, GREAT-ER has become increasingly complex due to new simulation and analyses features to fulfill the needs of different users such as scientists, authorities, (environmental agencies) and industry, and the demand for the tool has continuously increased. However, one of the major obstacles for widespread use of the model was the laborious preparation of the required data set for the catchment under investigation. Preparation of an executable database for a selected river basin demands a number of pre-processing steps, which has so far impeded broad application of the model by different users. This problem has been partly overcome since the freely available model version now comes along with a semi-automated data processing routine for catchment preparation, several tutorials, and an exemplary dataset of a hypothetical catchment with which users can set up a GREAT-ER database and familiarize themselves with its practical use.^1^ This forms a sufficient knowledge base for interested users to generate their own catchment database and proceed with the full version GREAT-ER 4.1.

A prerequisite for GREAT-ER simulations is assignment of realistic flow rates for average conditions (MQ), dry weather (MNQ), and the 50th percentile (Q50) to each river segment. There are numerous hydrological models (e.g., SWAT or NASIM) that can be used to estimate these data independently and import them into the GREAT-ER database. The GREAT-ER pre-processing provides an alternative semi-automated procedure to estimate river flow for each segment from spatially resolved runoff data for the whole catchment. Regardless how the MQ and MNQ values for each segment were estimated, they are calibrated against available gauging data before use.

Substance-specific parameters have to be entered manually into the respective fields of the database. Selected attributes in the database (e.g., number of people connected to a treatment plant) can be edited to keep it up-to-date.

### Case study simulations

**Fig. 1:**
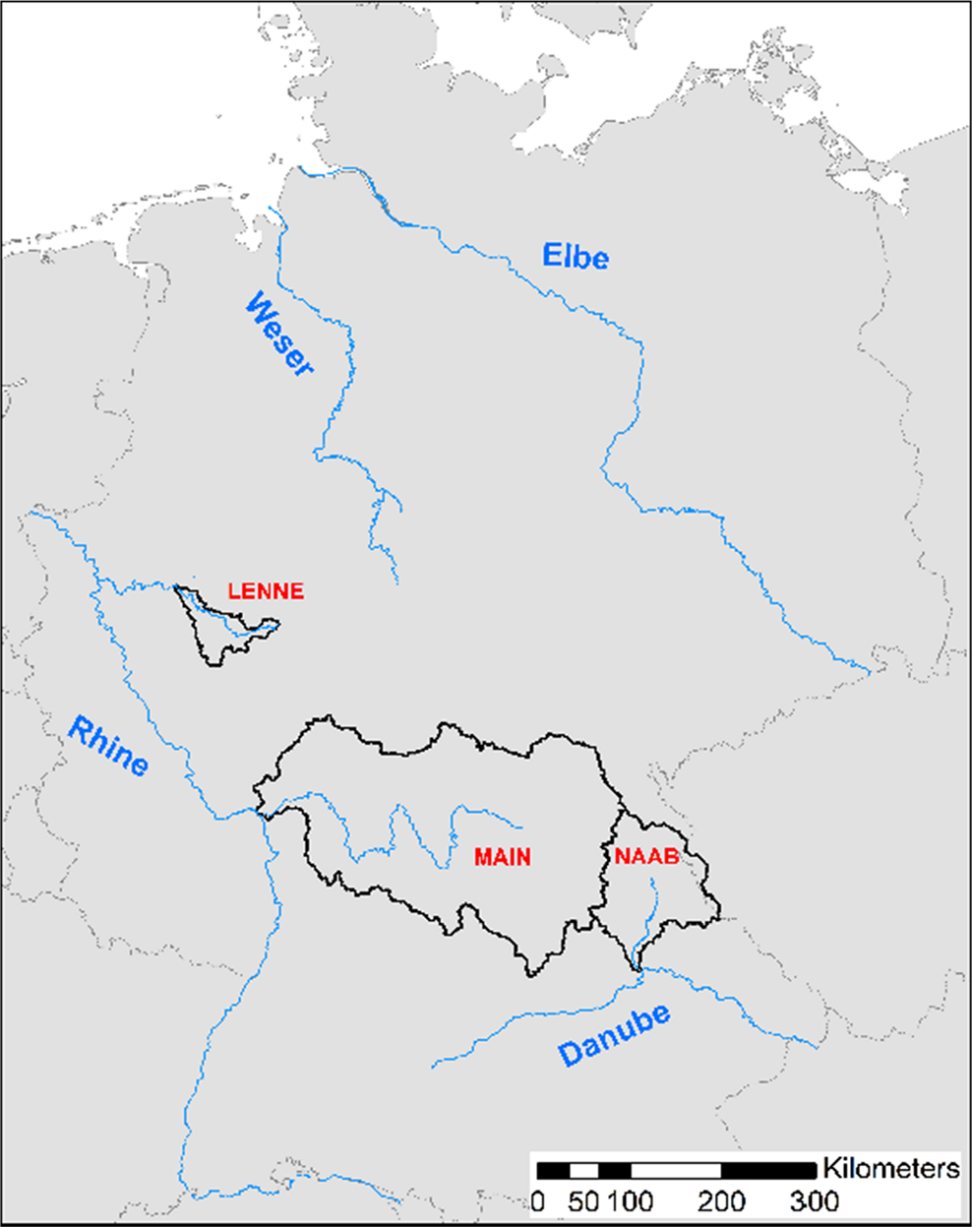
Location of the three German case study catchments: Main (1), Lenne (2), and Naab (3)

For the application of the model, three different pharmaceutical compounds in three German river basins of different size (see Fig. 1) have been simulated. The specific characteristics make them suitable to demonstrate some of the main benefits of the new model version for exposure (and risk) assessment. The selected substances were the antibiotic clarithromycin, the X-ray contrast agent iopamidol, and the natural hormone ethinylestradiol (EE2). All simulations were performed applying the implemented Monte Carlo simulation routine with 10,000 model realizations. All substance properties used for the model simulations are given in Table S1 in the SI. The location of the three catchments is shown in Fig. 1; basic characteristics are summarized in Table S2 in the SI.

## Results and discussion

### Simulation for clarithromycin in the Main catchment

**Fig. 2:**
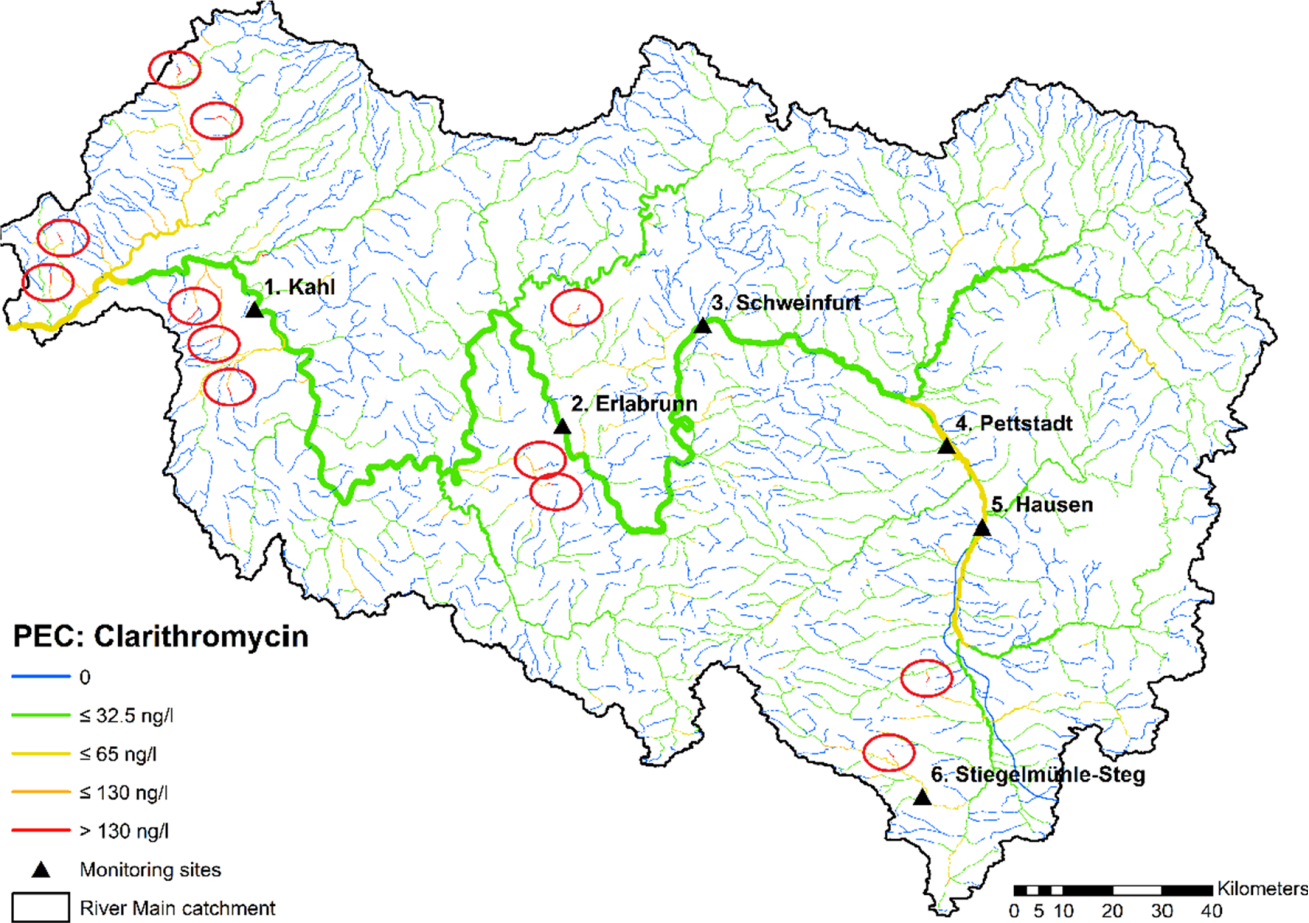
Color-coded map of average clarithromycin concentrations in the Main catchment predicted by GREAT-ER; hot spots (sites with highest concentrations) are highlighted by red circles; the six monitoring sites are marked as black triangles

Figure 2 shows predicted mean environmental concentrations (PEC), in the whole river basin in form of a color-coded map. This provides a quick overview of the spatial distribution of expected concentrations in the whole watershed and allows for easy identification of river segments with elevated concentrations. The environmental quality standard (EQS) of 130 ng/l for clarithromycin defined in the EU Water Framework Directive (WFD) (Carvalho et al. 2015) is only exceeded in a few small creeks with mean concentrations of up to 187 ng/l (red segments marked by circles in Fig. 2).

**Fig. 3:**
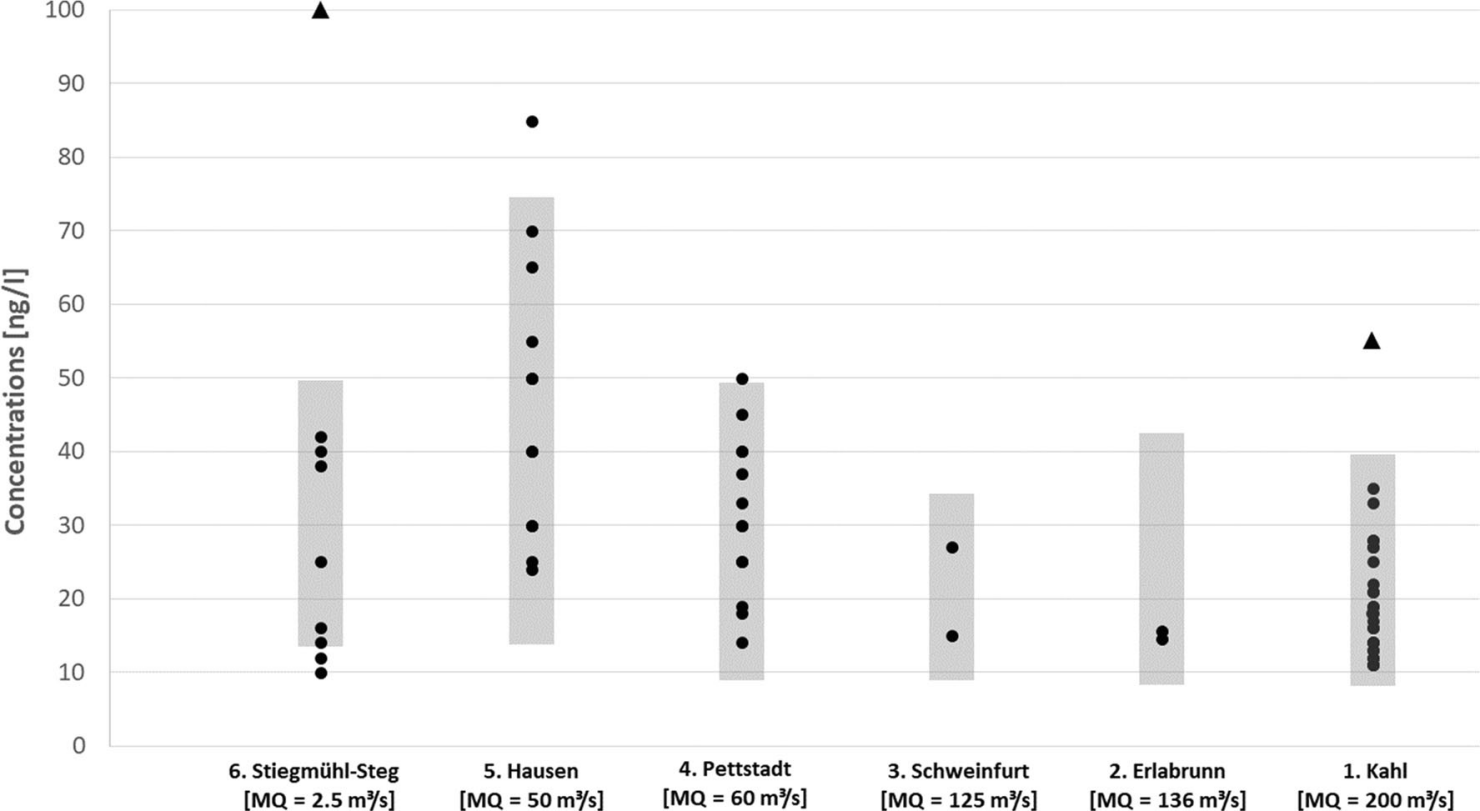
Comparison of clarithromycin measurements taken between 2010 and 2017 at the monitoring sites 1–6; sorted according to MQ; marked in grey the 10th-to-90th percent interval of 10,000 simulations runs. Two outliers according to Dean-Dixon test (*p* < 0.01) are marked with a triangle

The EU Commission Directive 2009/90/EC (EU 2009) specifies that an exceedance of EQS is incurred when the mean value of all measurements is above this threshold value. From the simulation results, it can be concluded that the majority of the river network will meet this regulatory criterion. Nevertheless, due to the large variability of river flows, concentrations may occasionally exceed the EQS at more sites even when mean values are below (Ort et al. 2010a). This can be investigated using the results of the probabilistic simulation. The probability distribution represents the expected variation of concentrations over time due to discharge fluctuations and input parameter uncertainties. Comparison with monitoring data was performed at six sites (locations shown in Fig. 2), for which multiple clarithromycin measurements were available (see Fig. 3). These sites cover a wide range of average river flow in the catchment going from 2 m^3^/s (site 6) up to more than 200 m^3^/s (site 1). Figure 3 demonstrates that the range spanned by the 10th and 90th percentile of simulated concentrations (displayed in grey) well represents the temporal variability of the monitoring data points at the six sites. At least 80% of the data points are within the respective probability range.

On top, the Dean-Dixon test (Dean and Dixon 1951) for small samples (*n* < 30) identified the two extremely high data points at sites 1 and 6, respectively, as outliers at a significance level of *p* = 0.01. The high concentration value of 100 ng/l at site 6 (Stiegmühl-Steg) may be explicable by specific temporal emissions due to the occurrence of combined sewage overflow (CSO) events. In the sampling period, intense precipitation in the area was recorded resulting in high flow rates approximately 50% above annual mean flow. It could well be that the water sample was affected by a recent CSO event having introduced large amounts of untreated wastewater. Consequently, emission loads of clarithromycin may have temporally jumped up even overcompensating the dilution effect by the higher flow rate.

### Simulation of iopamidol concentrations in the Lenne catchment

X-ray contrast agents such as iopamidol are applied exclusively in hospitals or private doctor’s offices for radiology. More than 90% of the applied dosage is excreted via urine within the first 24 h after administration (Duchin et al. 1986). In Switzerland, approximately 50% of X-ray contrast media are administered to stationary inpatients, and 75% of the dosage is already excreted in the urine within 4 h (Weissbrodt et al. 2009). Emissions from stationary treatments will surely enter the wastewater cycle at the location of medicinal treatment. We presume that additionally the first urinary excretion of treated non-stationary patients within the 4-h window will also occur at the treatment site so that 87.5% of the total administered dose was emitted there.

**Fig. 4:**
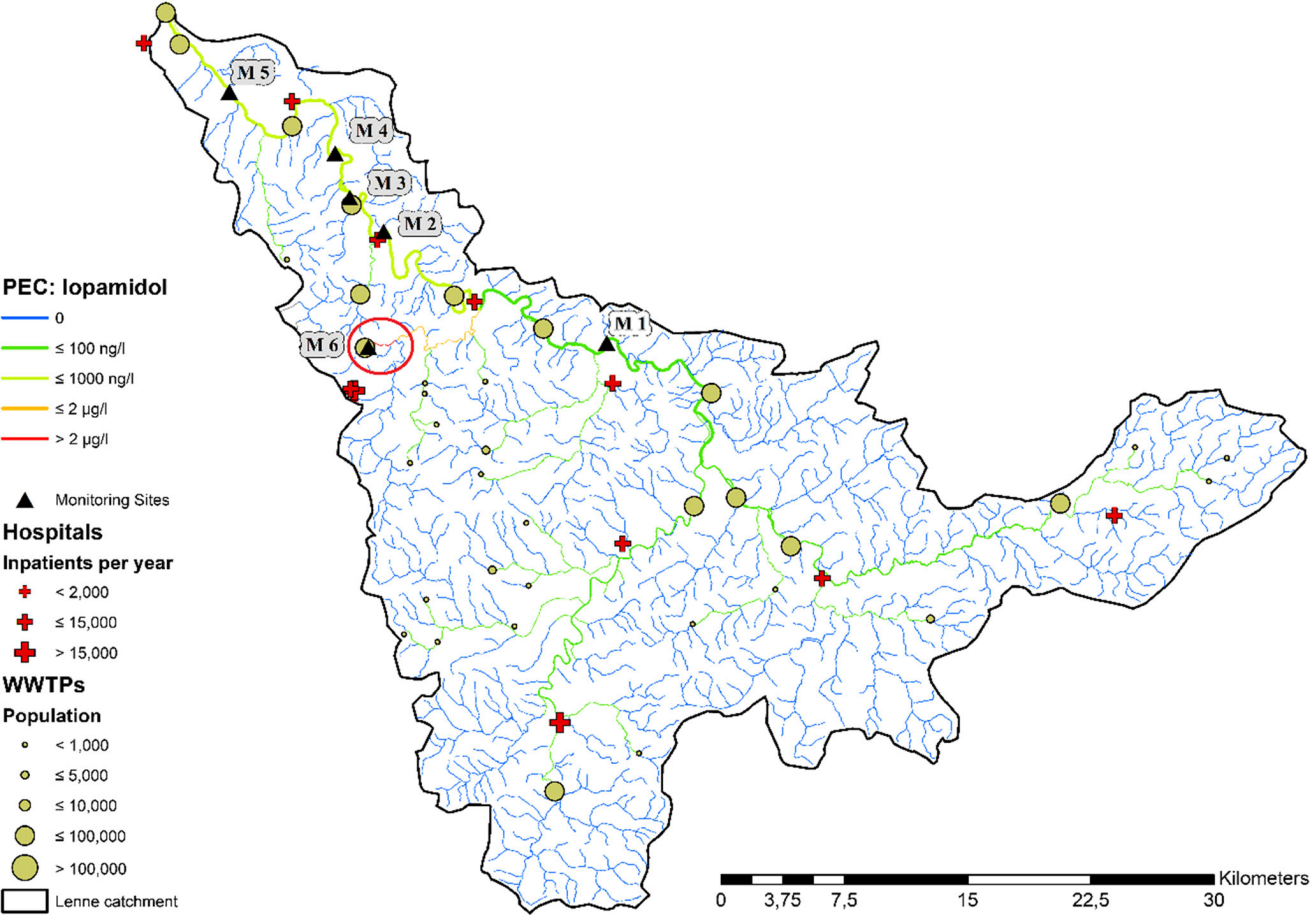
Color-coded map of the simulation results of a GREAT-ER model run (*n* = 10,000) for iopamidol in the Lenne catchment. The six monitoring sites (black triangles) are numbered from M1 to M6

For GREAT-ER model simulations, the iopamidol fraction excreted at the site of medicinal treatment (87.5%) was allocated to the eleven hospitals located in the Lenne catchment proportional to the total number of patients treated in the individual hospital. The resulting emission loads are then routed into the receiving sewage treatment plant, since hospitals are not directly emitting their wastewater into the river basin. The remaining emission fraction from prescriptions to non-stationary patients (12.5%) is still considered by the usual per capita approach according to the place of residence principle. This fraction represents the total iopamidol emission from patients after leaving the hospital or private doctor’s office and returning home. Figure 4 shows the result of the probabilistic simulation (*n* = 10,000) based on these assumptions (*standard scenario*).

**Fig. 5:**
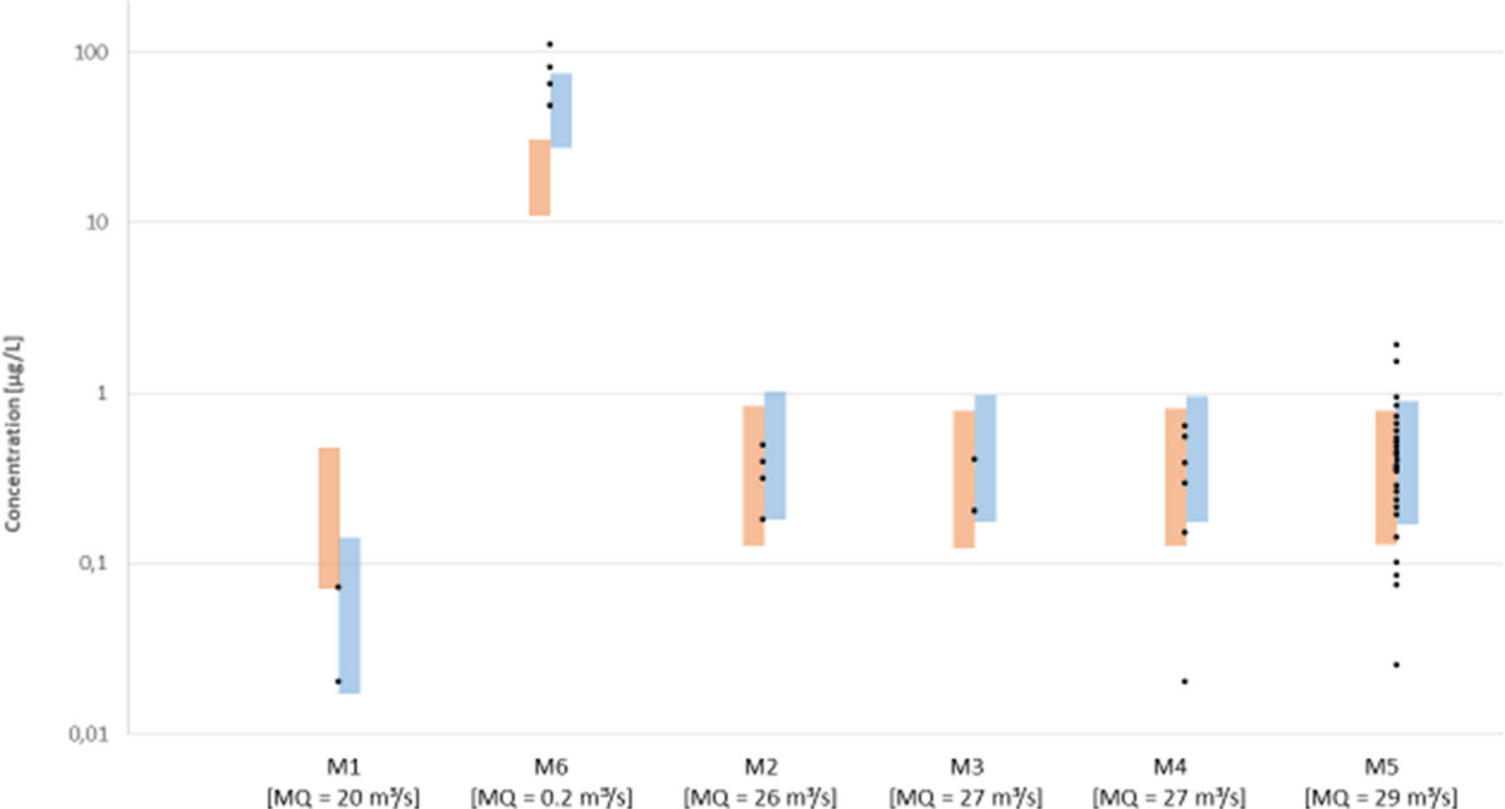
Comparison of the 10th-to-90th percent predicted concentration intervals of two probabilistic simulations (each *n* = 10,000). On the left (orange): interval for the standard scenario. On the right (blue): simulation with consideration of local hospital consumption patterns. Monitoring sites M1–M6 are arranged according the flow path of the Lenne; M6 is integrated according to the position of the tributary

The simulation results were compared with monitoring data for iopamidol at six locations (M1–M6) provided by the State Agency for Nature, Environment and Consumer Protection, North Rhine-Westphalia for the period from 2009 to 2015. Five sites are located along the Lenne River, while another one (M6) is in a small tributary, which enters the Lenne between M1 and M2. This site had been sampled on purpose to check the possible influence of the nearby hospital. Figure 5 (left) shows that the underlying model assumption of evenly distributed per patient consumption in hospitals (*standard scenario*) does not well reflect the overall situation of iopamidol concentrations in the Lenne basin. It turned out that the standard scenario underestimates the concentrations measured at M6, while data points at M1 were overestimated (see Fig. 5). At M6, even the 90th percentile of the simulation (31 μg/l) is below the four data points (46–110 μg/l) indicating stronger local influence of the nearby hospital. Further downstream (M2–M5), however, the results of the standard scenario simulation agree well with monitoring data.

It has already been shown that for some pharmaceuticals, the size of the hospitals alone could not always explain observed variations in hospital emissions (Kuroda et al. 2016; Kern et al. 2015). Thus, an overall per patient consumption without taking into account the presence or absence of specialized departments as proposed by Ort et al. (2010b) is not generally applicable. For more realistic local emission estimates, specific information such as department structure, stationary patients, and bed or dosage numbers should be considered if available. Since iopamidol is above all administered in specific radiology departments, the total number of patients may not be the best proxy for estimation of individual hospital emissions. Detailed review then revealed that there is only one hospital in the area, for which a radiology department is officially reported. Most likely, this hospital carries out the majority of radiological treatments with contrast agents relative to the total case numbers per year, as none of the other hospitals in the area is specialized in this field. Thus, for a second scenario, iopamidol emissions from hospitals were individually adjusted to increase the degree of realism in the model assumptions: The receiving WWTP of the respective hospital with radiology department was now loaded with an above average fraction of the iopamidol emissions, while the other hospitals’ contributions were decreased accordingly in order to keep the total emission constant. Before the adjustment, iopamidol emissions from hospitals were evenly distributed depending on their size (number of beds and patients). In the adjusted scenario, the single hospital with the radiology department is assumed responsible for 90% of the iopamidol hospital emissions (79% of overall emission). WWTP emissions from diffuse excretion away from the treatment location remained unchanged at 12.5% of total emissions, since reallocation of hospital contributions does not effect this number. Figure 5 shows simulated concentrations of iopamidol for the two scenarios compared to measured data.

The spatial redistribution of iopamidol hospital emissions in the model leads to a much better agreement with monitoring data as compared to the standard scenario at M1 and M6 (see Fig. 5, right), while further downstream (M2–M5), the previous good agreement persists. The model thus allows for consideration of local impacts of hospitals on surface water concentrations for specific pharmaceuticals, while the regional evaluation is only marginally affected. The analysis for iopamidol in the Lenne basin demonstrates that substances predominantly applied in large amounts at hospitals or private doctor’s offices experience a shift in their spatial concentration distribution that may locally be dependent on the presence or absence of specific medicinal departments.

### Simulation for ethinylestradiol in the Naab catchment

EE2 was chosen as exemplary compound, because it was on the first WFD watch list (2013) and remained part of the second edition (2018). Although extensive monitoring data have been already collected across Europe, the informative value of the data is still low due to the insufficient limit of quantification (LOQ) of the analytical methods. Only half of the responsible countries were able to quantify EE2 concentrations in the range of the EQS or below (Loos et al. 2018). This is where GREAT-ER simulations can be supportive, since for EE2, the model provides the sole possibility to get a comprehensive picture of the expected concentration range in a whole river basin even when concentrations are below the LOQ.

**Fig. 6:**
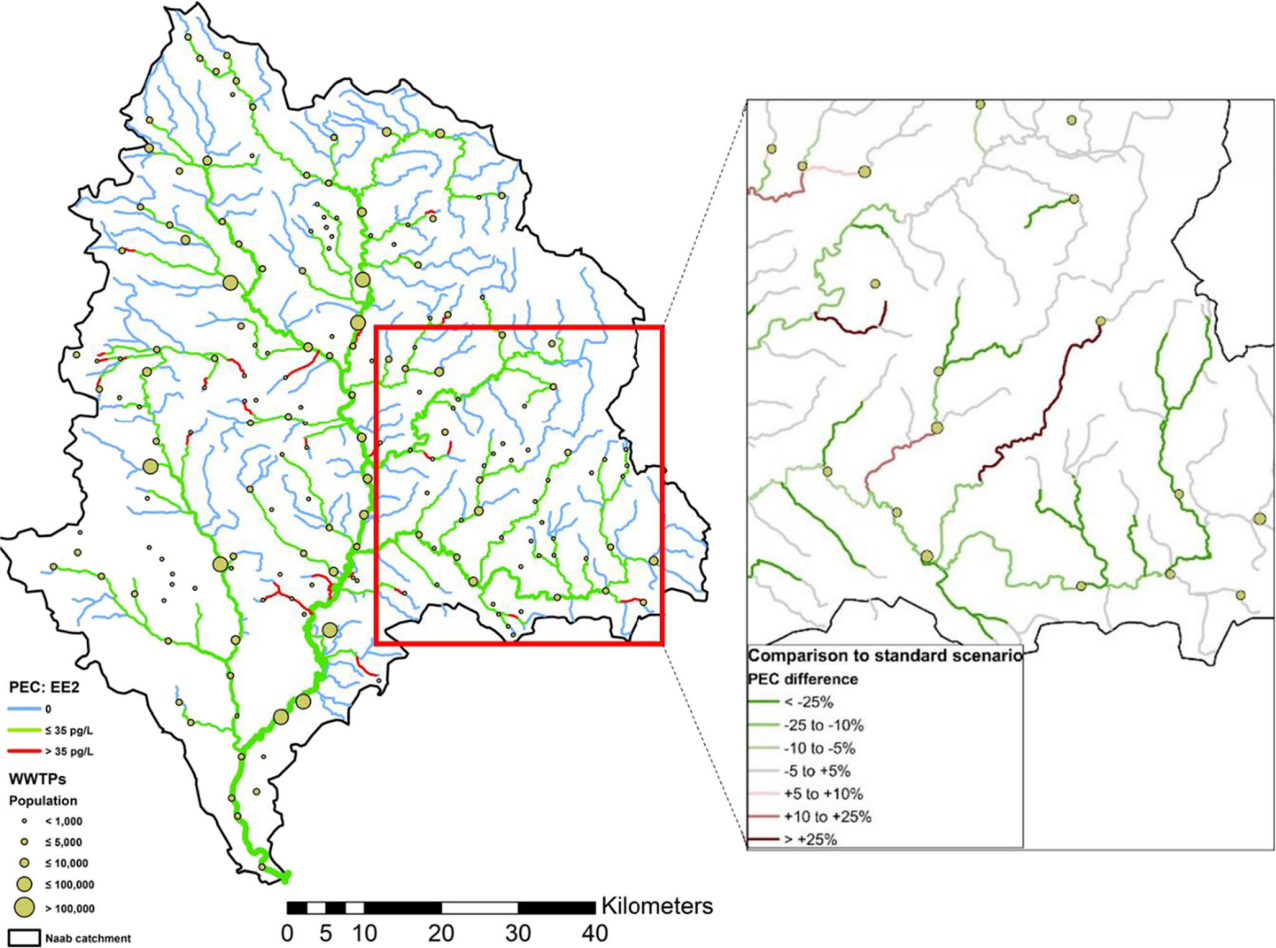
Left panel: PEC/EQS standard scenario. Right panel: relative change in PEC between action scenario and standard scenario for an exemplary area in the Naab catchment

The standard scenario representing the predicted status quo of average EE2 concentrations in the Naab catchment is displayed on the left-hand side of Fig. 6. The map reveals that EE2 concentrations in most of the river reaches do not exceed the currently proposed EQS of 35 pg/l (Loos et al. 2018). Moreover, only 65 km of the 2077 km flow length in the Naab basin downstream of WWTPs is predicted to exhibit EE2 concentrations detectable with the standard analytical procedures. Thus, comprehensive exposure assessment by monitoring cannot be achieved for EE2.

It is also seen that concentrations are highest in small creeks receiving wastewater from one of the 102 small treatment plants serving less than 1000 inhabitants (marked as small green dots in Fig. 6) with unfavorable dilution ratios. GREAT-ER provides a valuable tool to support authorities in decision-making by *a priori* simulation of the effect of mitigation measures. Therefore, we investigated the effect of a common strategy in the implementation process of the WFD in Germany, namely, re-routing of wastewater from these small WWTPs to the closest treatment plant with higher capacity (e.g., SMUV 2018; UM 2017). This closest distance boundary condition has been selected to minimize the length of additional sewer pipes for re-routing.

The result of this management scenario is shown in Fig. 6 (right) as relative comparison with the standard scenario. For river reaches displayed in green, PEC values in the action scenario are lower by at least 5% compared to the reference (improvement), while red river parts exhibit higher values (deterioration). Concentration changes of less than ±5% are regarded insignificant and thus marked gray.

In total, lower concentrations are predicted for 655 km flow length (32%) after re-routing, while only 91 km of the river system shows an increase in concentration of more than 5%. 6.1 km is now predicted to be above the EQS where there was no exceedance before, while 38.9 km is now below, resulting in a net relief of 32.8 km in sum. This is a direct consequence of the closest distance boundary condition. In the action scenario, redirection of wastewater does not always occur strictly downstream, because the closest larger treatment plant was sometimes located in another tributary’s sub-basin. In this case, water managers would have to evaluate different alternatives to find the best compromise between cost and effect. This case study demonstrates how the GREAT-ER model can support them to do so. In the first step, it provides information about the actual exposure situation (status quo) which allows for deciding whether there is a need for action at all. In the second step, the expected effect of selected measures can be evaluated in order to allow for implementing the most promising strategy taking into account cost-benefit considerations. In the case of EE2, GREAT-ER simulations predict mean concentrations in the Naab basin mostly below the current EQS so that immediate action does not seem to be necessary.

## Conclusions

The geo-referenced steady-state model GREAT-ER simulates the spatial concentration distribution under the assumption of steady state for specific boundary conditions. It was shown that probabilistic simulations considering natural variability of river flow and/or uncertainty of model parameters well predict the expected range of concentrations. We conclude that exposure assessment in river basins should not solely rely on a restricted number of monitoring data but make use of the complementary GREAT-ER model approach.

However, the general assumption of more or less evenly distributed emission patterns does not hold true for pharmaceuticals administered in large fractions in hospitals or private doctors’ offices. While this does not largely affect exposure assessment on the regional scale, local assessment may fail for such compounds if the flow path of hospital wastewaters is not explicitly considered in the model representation.

Exposure and risk assessment for micropollutants at low concentrations in the range of the limit of detection constitutes a particular challenge. A prominent example for this dilemma is EE2 due to its low exposure concentrations and the low EQS value proposed. While in such cases monitoring alone is not sufficient for basin-wide exposure assessment, this can be achieved with the support of the GREAT-ER model.

An essential part of the GREAT-ER software is the ability to create and analyze specific action scenarios. These features can be used for *a priori* assessment of measures on the catchment scale. For example, re-routing of wastewater from decentralized small WWTPs to larger ones has been shown to provide an option for improvement of the water quality in small creeks with unfavorable dilution factors.

This may be all the more important as the EU recently has run so-called “fitness checks,” assessing whether EU Directives are fit for purpose by examining their performance. The WFD was checked aside the Environmental Quality Standards Directive, the Groundwater Directive, and the Floods Directive (EU 2019). While this fitness check states that in Germany, the implementation of the WFD has led to an improvement of the state of numerous waters and the knowledge on pollutant loads and water quality could be increased considerably, it adds that most of Germany’s water bodies will not achieve the 2027 targets (Vermeulen et al. 2019). We conclude that complimentary use of targeted monitoring and geo-referenced modeling constitutes a promising option to save time and money while completing these tasks.

## Data Availability

A basic version of the GREAT-ER 4 model along with tutorials is available on registration at www.usf.uni-osnabrueck.de/en/forschung/applied_systems_science/great_er_project. All external monitoring data analyzed during the study are available from the corresponding author on reasonable request.

## Supporting information

ESM 1 (DOCX 28 kb)

## Footnotes

1 Program details available at: www.usf.uni-osnabrueck.de/en/forschung/applied_systems_science/great_er_project
