# Supplementary material for: Geo-referenced simulation of pharmaceuticals in whole watersheds: application of GREAT-ER 4.1 in Germany
Source: Environ Sci Pollut Res Int. 2021 Jan 7;28(17):21926–35. doi: 10.1007/s11356-020-12189-7 (PMC8106600; doi:10.1007/s11356-020-12189-7)
Supplement: ESM 1 — (DOCX 28 kb) [file 11356_2020_12189_MOESM1_ESM.docx]

Supporting Material to the manuscript

for Environmental Science and Pollution Research

entitled

Geo-referenced simulation of pharmaceuticals in whole watersheds - Application of GREAT-ER 4.1 in Germany

authored by

Volker Lämmchen, Gunnar Niebaum, Jürgen Berlekamp, Jörg Klasmeier∗

Institute of Environmental Systems Research, Barbarastr. 12, 49076 Osnabrück, Germany

Contents:

Table S1: Substance properties used for simulations

Table S2: Main characteristics of investigated river basins

References

**Table S1**: Substance properties for model simulations

|  |  | **Clarithromycin** | **Iopamidol** | **Ethinylestradiol** | **Reference** | | |
| --- | --- | --- | --- | --- | --- | --- | --- |
|  |  | **I** | **II** | **III** | **I** | **II** | **III** |
| ***Phys. chem. properties*** | Unit |  |  |  |  |  |  |
| Molar mass | g/mol | 747.96 | 777.08 | 296.1 | [24] | [21] | [11] |
| log Kow  Water solubility  pKa | mg/l | 3.16  0.336  8.95 | - 2.42  120  10.7 | 3.67  11.3  10.4 | [24]  [24]  [24] | [21]  [21]  [21] | [11]  [26]  [26] |
| **WWTP removal**  Lagoon  Wetland  Biofilm  Activated Sludge | %  %  %  % | 30.5  30.5  44  44 | < 10  < 10  60 – 80  35 | >70  >70  87  87 | [20]  [20]  -  [9], [19], [24] | [10]  [10]  [6]  [10] | [8]  [8]  [4]  [4], [15] |
| ***River removal***  Half-life  Model assumption  Near surface photolysis  Kd river | d  1/h  1/h  L/kg | 0.001  335 | > 44  6.6e-4  -  - | 5,50e-03  140 | [20]  [1] | [17]  [23]  -  - | [16]  [16] |
| ***Consumption***  Per-capita consumption  Hospital fraction | kg/(Inhabitant*a)  % | 1.28e-04  15.2 | 6.6e-04  87.5 | 5,596e-07  - | [22]  [5] | [13]  [7] | IMS, 2014  - |
| ***Excretion*** | % | 30 | 87.5 | 40 | [2], [19] | [7] | [14] |
| ***EQS*** | ng/l | 130 | - | 0.35 | [3] | - | [3] |

**Table S2**: Main characteristics of investigated river basins

|  |  | **Main** | **Lenne** | **Naab** |
| --- | --- | --- | --- | --- |
|  | Unit |  |  |  |
| Size | [km²] | 27,250 | 1,352 | 5,225 |
| Connected inhabitants |  | ~ 3,800,000 | ~ 380,000 | ~ 500,000 |
| Number of WWTPs |  | 848 | 36 | 192 |
| Flow length of the main stream | [km] | 527 | 129 | 98 |
| Cumulated length of the simulated river network | [km] | 10,273 | 5,156 | 2,077 |
| MQ-Discharge at the outlet point | [m³/s] | ~250 | ~ 28 | ~ 50 |

## References

1. Azuma, T., Ishida, M., Hisamatsu, K., Yunoki, A., Otomo, K., Kunitou, M., Shimizu, M., Hosomaru, K., Mikata, S., Mino, Y., 2017. Fate of new three anti-influenza drugs and one prodrug in the water environment. Chemosphere 169, 550–557.
2. Baumann, M., Weiss, K., Maletzki, D., Schüssler, W., Schudoma, D., Kopf, W., 2015. Chemosphere Aquatic toxicity of the macrolide antibiotic clarithromycin and its metabolites. Chemosphere 120, 192–198.
3. Carvalho, R.N., Ceriani, L., Ippolito, A., 2015. Development of the first Watch List under the Environmental Quality Standards Directive water policy. Report EUR 27142 EN.
4. Clara M., Strenn B, Ausserleiter M, Kreuzinger N., 2004. Comparison of the behaviour of selected micropollutants in a membrane bioreactor and a conventional wastewater treatment plant.Water Sci Technol 50: 29–36.
5. Coutu, S., Rossi, L., Barry, D.A., Rudaz, S., Vernaz, N., 2013. Temporal Variability of Antibiotics Fluxes in Wastewater and Contribution from Hospitals. PLOS ONE 8(1): e53592.
6. Escolà Casas, M., Chhetri, R. K., Ooi, G., Hansen, K. M. S., Litty, K., Christensson, M., … Bester, K. (2015). Biodegradation of pharmaceuticals in hospital wastewater by a hybrid biofilm and activated sludge system (Hybas). Science of The Total Environment, 530-531, 383–392.
7. Duchin, K. L., Drayer, B. P., Ross, M., Allen, S., Frantz, M., 1986. Pharmaocokinetics of iopamidol after intrathecal administration in humans. Am J Neuroradiol; 7: 895-8.
8. Froehner S, Piccioni W, Machado KS, Aisse MM, 2011. Removal capacity of caffeine, hormones, and bisphenol by aerobic and anaerobic sewage treatment. Water Air Soil Pollut 216: 463–71.
9. Göbel, A., Thomsen, A., McArdell, C., Joss, A., Giger, W., 2005. Occurrence and Sorption Behavior of Sulfonamides, Macrolides, and Trimethoprim in Activated Sludge Treatment. Environ. Sci. Technol. 39, 3981-3989.
10. Götz, C., Bergmann, S., Ort, C., Singer, H., Kase, R., 2012. Mikroschadstoffe aus kommunalem Abwasser - Stoffflussmodellierung, Situationsanalyse und Reduktionspotenziale für Nordrhein-Westfalen, Studie im Auftrag des Ministeriums für Klimaschutz, Umwelt, Landwirtschaft, Natur- und Verbraucherschutz Nordrhein-Westfalen (MKULNV).
11. Hansch, C., Leo, A., D. Hoekman, 1995. Exploring QSAR - Hydrophobic, Electronic, and Steric Constants. Washington, DC: American Chemical Society., p. 168.
12. Hijosa-Valsero, M., Fink, G., Schlüsener, M.P., Sidrach-Cardona, R., Martín-Villacorta, J., Ternes, T., Bécares, E., 2011. Removal of antibiotics from urban wastewater by constructed wetland optimization. Chemosphere 83, 713–719.

1. Internationale Kommission zum Schutz des Rheins (IKSR), 2010. Auswertungsbericht Röntgenkontrastmittel. Koblenz.
2. Johnson, A. C., Williams, R. J., 2004. A model to estimate influent and effluent concentrations of estradiol, estrone, and ethinylestradiol at sewage treatment works. Environmental Science & Technology, 38(13), 3649-3658.
3. Joss A, Andersen H, Ternes T, Richle PR, Siegrist H., 2004. Removal of estrogens in municipal wastewater treatment under aerobic and anaerobic conditions: consequences for

plant optimization. Environ Sci Technol 38: 3047–55.

1. Jürgens, M. D., Holthaus, K. I. E., Johnson, A. C., Smith, J. J. L., Hetheridge, M., Williams,R. J., 2002. The potential for estradiol and ethinylestradiol degradation in English rivers. Environmental Toxicology and Chemistry 21(3): 480-488.
2. Kormos,J.L., Schulz,M.; Kohler,H.-P.; Ternes,T.A. (2010): Biotransformation of selected iodinated X-ray Contrast Media and Characterization of Microbial Transformation Pathways. *Environ. Sci. Technol*. 44(13), 4998-5007.
3. Kormos, J.L., Schulz, M., Ternes, T.A., 2011. Occurrence of Iodinated X-ray Contrast Media and Their Biotransformation Products in the Urban Water Cycle 8723–8732.
4. Kümmerer, K., Henninger, A., 2003. Promoting resistance by the emission of

antibiotics from hospitals and households into effluent. Clin. Microbiol. Infect. 9, 1203–1214.

1. Nakada, N., Shinohara, H., Murata, A., Kiri, K., Managaki, S., Sato, N., Takada, H., 2007. Removal of selected pharmaceuticals and personal care products (PPCPs) and endocrine-disrupting chemicals (EDCs) during sand filtration and ozonation at a municipal sewage treatment plant. Water Research 41, 4373-4382.
2. O'Neil, M.J. (ed.), 2006. The Merck Index - An Encyclopedia of Chemicals, Drugs, and Biologicals. Whitehouse Station, NJ: Merck and Co., Inc., p. 879.
3. Schwabe, U., Paffrath, D., Ludwig, W.-D., Klauber, J. (Hrsg.), 2017. Arzneiverordnungs-Report 2017 - Aktuelle Daten, Kosten, Trends und Kommentare. Springer Verlag. Berlin.
4. Ternes, T. A., Hirsch, R., 2000. Occurrence and behavior of X-ray contrast media in sewage facilities and the aquatic environment. *Environ. Sci. Technol.* 34(13), 2741-2748.
5. Ternes, T.A., Bonerz, M., Herrmann, N,; Teiser, B., Andersen, H.R., 2007. Irrigation of treated wastewater in Braunschweig, Germany: An option to remove pharmaceuticals and musk fragrances. Chemosphere 66, 894-904.
6. Vione, D., Feitosa-Felizzola, J., Minero, C., Chiron, S., Giuria, V. Pietro, Analitica, C., 2009. Phototransformation of selected human-used macrolides in surface water : Kinetics , model predictions and degradation pathways. Water Res. 43, 1959–1967.
7. Yalkowsky, S.H., He, Yan., 2003. Handbook of Aqueous Solubility Data: An Extensive Compilation of Aqueous Solubility Data for Organic Compounds Extracted from the AQUASOL dATAbASE. CRC Press LLC, Boca Raton, FL., p. 1158
